# Label-free *in situ* imaging of oil body dynamics and chemistry in germination

**DOI:** 10.1098/rsif.2016.0677

**Published:** 2016-10

**Authors:** Gustav Waschatko, Nils Billecke, Sascha Schwendy, Henriette Jaurich, Mischa Bonn, Thomas A. Vilgis, Sapun H. Parekh

**Affiliations:** 1 Department of Molecular Spectroscopy, Max Planck Institute for Polymer Research, Mainz, Germany; 2 Department of Polymer Theory, Max Planck Institute for Polymer Research, Mainz, Germany

**Keywords:** Raman imaging, coherent Raman microscopy, plant lipids, emulsions, oil bodies, lipid droplets

## Abstract

Plant oleosomes are uniquely emulsified lipid reservoirs that serve as the primary energy source during seed germination. These oil bodies undergo significant changes regarding their size, composition and structure during normal seedling development; however, a detailed characterization of these oil body dynamics, which critically affect oil body extractability and nutritional value, has remained challenging because of a limited ability to monitor oil body location and composition during germination *in situ*. Here, we demonstrate via *in situ*, label-free imaging that oil bodies are highly dynamic intracellular organelles that are morphologically and biochemically remodelled extensively during germination. Label-free, coherent Raman microscopy (CRM) combined with bulk biochemical measurements revealed the temporal and spatial regulation of oil bodies in native soya bean cotyledons during the first eight days of germination. Oil bodies undergo a cycle of growth and shrinkage that is paralleled by lipid and protein compositional changes. Specifically, the total protein concentration associated with oil bodies increases in the first phase of germination and subsequently decreases. Lipids contained within the oil bodies change in saturation and chain length during germination. Our results show that CRM is a well-suited platform to monitor *in situ* lipid dynamics and local chemistry and that oil bodies are actively remodelled during germination. This underscores the dynamic role of lipid reservoirs in plant development.

## 1. Introduction

Oleosomes are subcellular lipid reservoirs (oil bodies) in all plant seeds that are found primarily in their cotyledons (embryonic seed leaves) and embryonic axis (seedling's root) [1]. Oil bodies—filled with mostly triacylglycerols (TAGs) and to a lesser extent sterol esters, diacylglycerols, monoacylglycerols and free fatty acids (FFAs)—are plant analogues to classical mammalian lipid droplets. They provide (a source of) energy—in the form of FFAs for β-oxidation in neighbouring glyoxysomes (peroxisomes)—during initial seed germination [2] and lipids for new cell and organelle membranes. The structure of oil bodies also resembles lipid droplets. The oil core is surrounded by a phospholipid monolayer and associated regulatory proteins; however, oleosomes possess additional emulsifying proteins called oleosins [3,4]. Oleosins, which contain both strongly hydrophobic and strongly hydrophilic domains, are highly effective oil body emulsifiers, and, accordingly, suppression of oleosin production via RNAi or complete removal by knockout [5,6] results in larger oil bodies and altered availability of lipid fuel for plant germination [7]. Moreover, oil bodies in seed maturation have been shown to be synthesized via endoplasmic reticulum budding during seed maturation—similar to mammalian lipid droplets—and budding requires oleosins to maintain the appropriately small oil bodies in the mature seed [8–11]. Indeed, it has further been shown that, following *in vitro* proteolysis of surface oleosins, oil bodies coalesced, as is expected for lipid droplets with only a phospholipid monolayer [12,13].

Owing to the initial absence of photosynthesis in germination, nearly all energy for initial development in plants comes from lipids via lipolysis of TAGs by surface-bound lipases, β-oxidation in glyoxysomes and catabolism in mitochondria [3]. As a result, the density of oleosomes, and correspondingly of oleosins, is initially quite high: for instance, oleosins constitute nearly 10% of the total protein mass in *Arabidopsis thaliana* seeds [11]. The high levels of oleosin can be understood from its important role as an emulsifier, helping to maintain small oil bodies with a high surface-to-volume ratio for augmented lipolysis by surface-localized lipases [14,15].

Although previous studies have shown that oleosin disappears from oil bodies during germination [16,17] and that oil bodies fuse when oleosin is genetically suppressed [5,6], it is unknown if oil bodies grow or shrink during unperturbed, native germination and how this correlates to oleosin levels. Recent work has shown that oleosins are degraded prior to lipid mobilization from oil bodies via a ubiquitination–proteasome pathway. Protease inhibitors reduced lipid consumption and led to depots of oleosin aggregates in *Arapibodpsis thaliana* [17]. This strongly suggests that oleosin degradation is connected with lipid mobilization; however, if a similar oleosin degradation pathway exists in soya beans—and how this might affect oil body composition—is not known.

While providing substrates for eventual ATP production is undoubtedly a primary function of oil bodies, such intracellular lipid depots have attracted increasing attention over the past decades, owing to the discovery of their functional and dynamic behaviour in many organisms [18,19]. Indeed, lipid droplet regulation is closely related to metabolic and developmental disorders in mammals, such as type 2 diabetes [18], and protection against fungal pathogens in plants [20]. Because of the multi-faceted role that oil bodies (and lipid droplets) play (as energy sources, lipotoxicity protectors and protein captors), insights into the changes in the morphology, biochemistry and protein coating of oil bodies under native physiological conditions are essential for understanding development.

Imaging of oil bodies in plants is challenging. The use of typical fluorescent probes is potentially problematic due to the relatively small size of lipids compared with typical fluorescent probes (approx. 2 : 1 lipid : fluorophore in weight). Indeed, such probes have been shown to perturb native lipid behaviour [21,22]. Furthermore, an additional challenge in plants exists because of the cell wall, which is largely impermeable to traditional labelling approaches with BODIPY, Nile red and oil red O staining. These complications make fluorescence imaging of lipids challenging in fixed tissues, if not impossible *in vivo* in plants.

Classically, evaluation of lipid biochemistry in tissues involves extraction and subsequent gas chromatography to quantitatively determine the amount of each individual lipid subtype present in a sample [23]. While extremely accurate for chemical identification, this process compromises any spatial information of microscopic organization. More recently, matrix-assisted laser desorption ionization–imaging mass spectrometry (MALDI-IMS) and magnetic resonance imaging (MRI) of lipids have emerged as attractive methods that offer better spatial localization without sacrificing chemical specificity. MALDI-IMS allows detection with high sensitivities (femto- to atto-molar) in a local region of the sample (approx. 3–10 µm voxel size) for a large range of masses (from approx. 100 Da to approx. 300 kDa) [24,25]. Indeed using MALDI-IMS, it has been shown that lipids in different parts of germinating seeds have different compositions, which underscores area-specific development of different organelles within the same seed [26]. However, achieving such high resolution requires careful matrix embedding and sample preparation, which may affect tissue structure and localization of biomolecules. Furthermore, the spatial resolution is insufficient to interrogate individual oil bodies (0.05–3 µm diameter) at this time [24,27].

An alternative approach for local lipid analysis is chemical imaging via nuclear magnetic resonance (NMR) or vibrational microscopy, which requires little to no sample preparation (fixation at most). Lipids are readily identifiable with both techniques via their intrinsic molecular spectroscopic signatures and thus require no labelling. NMR-based imaging has the ability to image specific signals from lipids, water or other metabolites as well as acquire hyperspectral data—an NMR spectrum at each voxel—in macroscopic specimens with voxel sizes as small as approximately 10 × 10 × 100 µm^3^ [28]. This gives a unique overview of lipid organization in an entire specimen and has been used to show a gradient in lipids within soya bean cotyledons, with higher TAG content in the central and adaxial (inner) region than in the abaxial (outer) region [29,30]. NMR imaging is non-invasive, label-free, chemically specific and offers spatial resolution on the micro-scale—comparable to MALDI-IMS; however, it is similarly not well suited to image individual oil bodies or body clusters in cells because of the limited spatial resolution. Furthermore, acquisition times can be relatively long (approx. 10 h) for high-quality hyperspectral datasets [28].

Raman scattering also derives contrast from intrinsic molecular vibrations in the sample, e.g. from the abundant CH_2_ groups that are strongly present in lipid acyl chains. Furthermore, because of the chemical specificity of this approach to functional groups, similar to NMR, it is possible to image entire macromolecular species, e.g. all lipids, all proteins, all nucleic acids, etc., within a sample. Since the excitation for Raman scattering is visible light, the diffraction-limited optical resolution, approximately 250–300 nm in plane, offers the ability to localize individual (or small clusters of) oil bodies within single cells as an imaging modality. Therefore, chemical imaging based on Raman scattering potentially provides a non-invasive molecular fingerprint of lipids within oil bodies *in situ*, which is, to the best of our knowledge, not possible with any other method.

In this work, we use coherent Raman microscopy (CRM)—specifically coherent anti-Stokes Raman scattering (CARS)—to image lipids *in situ* and *in vivo* without labels and quantify biochemical changes of these oil bodies during germination. CARS effectively boosts the Raman signal strength by resonant enhancement by up to six orders of magnitude and circumvents challenges with autofluorescence [31–33]. Our results demonstrate that oil body size and spatial distribution are dynamic and are paralleled by changes in lipid composition as germination proceeds. CRM, together with additional biochemical analyses, shows that lipid-associated proteins, and specifically oleosins, disappear as larger oil bodies appear in the cotyledon. Interestingly, we also observe that oil bodies again decrease in size after initially growing. These results show that oil bodies are not static depots during germination but rather are actively remodelled to transform from oleosin-coated oil bodies to non-oleosin-coated oil bodies [34] with dynamic lipid species contained within them.

## 2. Material and methods

### 2.1. Soya bean germination

Ten grams of soya beans (Glycine max; Davert GmbH, Ascheberg, Germany) were assembled in one line on a sheet of aluminium foil in between four layers of wet Kimtech science precision wipes 21 × 11 cm (Kimberly Clark, Roswell, GA, USA). They were covered with a second piece of aluminium foil (30 × 140 cm in total) and dipped in deionized water (18 cm distance from bean to water) to imbibe the beans and continually moisturize them during growth [35]. Soya beans were incubated at 25°C in the closed aluminium foil up to day 5, after which their cotyledons were released by removing the upper part of the foil. The exposed seedlings were returned to the water and cultivated under the same conditions up to day 9. For each day, representative soya beans were selected and documented by total plant size and weight (electronic supplementary material, figure S1) based on a set of criteria: root size ±25% (for days 2–6), colour of cotyledons, secondary roots and primary leaves (for days 6–8).

### 2.2. Oil body purification

In total, 11–30 soya beans matching the above-defined germination stage criteria were selected at the specified day after imbibition and ground to extract oil bodies as described previously [36,37]. Briefly, the selected beans were placed into 100 ml water and ground in a Thermomix TM31 (Vorwerk, Wuppertal, Germany) at the highest speed (10 200 r.p.m.) for 60 s. This slurry was filtered through two layers of 21 × 11 cm Kimtech science precision wipes to obtain raw soya milk. Twenty-five per cent sucrose (w/w) was added to the raw soya milk and the pH was adjusted to 11.0 with 1 mol l^−1^ sodium hydroxide to remove weakly bound proteins, such as conglycinin, glycinin, allergenic proteins and oleosin proteases. This made subsequent Bradford measurements more specific for only strongly bound proteins such as oleosins, caloleosins and steroleosins [16,36,38]. We note that using high alkaline pH reduces the total amount of oleosins compared with neutral extraction [39], which could cause a systematic offset for subsequent oil body size measurements from laser diffraction and Bradford measurements of protein quantity. The solution was poured into 50 ml centrifuge tubes and centrifuged in a Multifuge X1R (Thermo Heraeus, Hanau, Germany) at 15 000*g* and 4°C for 3 h. The resulting floating fractions (cream layer, fat pad and oil bodies) were removed with a small spoon and re-suspended in 15 ml of deionized water.

### 2.3. Analysis of proteins associated with oil bodies

After the first centrifugation step (see Oil body purification), sucrose (20% w/w) was added and the pH was adjusted to pH 11. The mixture was centrifuged again (15 000*g*, 4°C, 3 h). This sequence of steps was repeated once more to remove any residual storage proteins in the oil bodies [37] and to denature the proteases Bd 30 K and P34 [16]. Finally, the washed oil bodies were collected with a spoon, dispersed in 10 ml phosphate-buffered saline (PBS), and stored at 4°C for protein and lipid analysis. Prior to any lipid or protein analysis, the dry weight of the purified oil bodies was determined using a precision moisture balance HR83 (Mettler Toledo, Geissen, Germany).

Protein quantification of the oil bodies was determined using a standard Bradford assay with the Bradford reagent B6916 (Sigma, Steinheim, Germany) by using the same dry weight of oil bodies. For detailed protein analysis, sodium dodecyl sulfate–polyacrylamide gel electrophoresis (SDS-PAGE) was performed with the NuPAGE-System (Life Technologies, Darmstadt, Germany). A 10% NuPAGE resolving gel was used with the manufacturer's instructions but without heating the samples (10 mg ml^−1^ purified oil bodies) to avoid lipid smear. Instead, they were solubilized overnight at room temperature with the NuPageLDS Sample Buffer and the NuPageLDS Reducing Agent. Protein bands were visualized with Coomassie staining, G-250 SimplyBlue SafeStain (Life Technologies, Darmstadt, Germany).

### 2.4. Fixation and slicing of tissue sections

In fixed samples, cotyledons of representative germinated soya beans were cut into quarters and fixed with 4% paraformaldehyde with 150 mM sucrose at 4°C overnight. These samples were subsequently flash frozen in liquid nitrogen and sliced with a cryotome into 15 µm thick slices at −20°C with an MTC cryostat (SLEE Medical, Mainz, Germany). Care was taken to obtain slices from a location 3–5 mm from the embryo. These slices were mounted on glass slides, sandwiched in 40 µl PBS by attachment of a no. 1 coverslip, and sealed with nail polish prior to imaging. Representative Z-stacks at the specified Raman shift were acquired at each day from the adaxial (flat part), centre and abaxial (curved) regions of the cotyledon cross-section (see the electronic supplementary material, figure S2) [30].

### 2.5. Coherent anti-Stokes Raman scattering microscopy

CARS was used to image oil bodies *in situ* in soya bean cotyledons. CARS is a nonlinear Raman microscopy method involving three-photon interaction: pump (*ω*_pump_), Stokes (*ω*_Stokes_) and probe (*ω*_probe_) photons to generate a fourth anti-Stokes photon (*ω*_anti-Stokes_ = *ω*_pump_ – *ω*_Stokes_ + *ω*_probe_), where *ω*_pump_, *ω*_Stokes_, *ω*_probe_ and *ω*_anti-Stokes_ are the energies of the pump, Stokes, probe and anti-Stokes photons, respectively. The Raman shift Δ_Raman_ = *ω*_pump_ – *ω*_Stokes_ was set to 2845 cm^−1^ for all images in this study. Laser excitation was provided by picosecond pulses at 1064 nm and 817 nm (APE, Berlin, Germany). CARS microscopy was performed on an SP 5 II TCS CARS microscope equipped with transmitted and reflected light photomultiplier tubes (Leica, Mannheim, Germany) and a 1.49 numerical aperture (NA), 60× oil-immersion objective (Nikon, Tokyo, Japan). The laser power varied by 4% over the entire imaging period (three weeks), while all other imaging and scanning parameters were held constant. Oil bodies were imaged in the transmitted photomultiplier tube with an additional 660 nm bandpass filter (full-width half-maximum of 8 nm) (Photomed GmbH, Seefeld, Germany) in addition to the standard Leica CARS 2000 filter set. Samples were imaged either as fixed tissues or as live beans.

### 2.6. Live imaging of a single cotyledon

Three-day-old soya beans were cut open at a position 3–5 mm from the embryo and were placed with the cut face on a glass-bottomed dish (WillCo-dish^®^) touching blocks of agarose with the root. The cotyledons were fixed with glue and the dish was closed on two sides. Images were acquired at a line rate of 200 Hz, with 1024 × 1024 lateral pixels in each field of view, and 28–33 axial slices. The pixel sizes were 0.252 µm (lateral), 1.01 µm (axial).

### 2.7. Quantification of oil bodies in CARS images

Z-stacks were acquired at a line rate of 200 Hz, with 1024 × 1024 lateral pixels in each field of view. The pixel sizes were 0.168 µm (lateral) and 0.545 µm (axial). Three beans were imaged for each cotyledon region for each day. Sub-stacks from the centre of each Z-stack, consisting of six images, were selected with ImageJ. These sub-stacks were maximum intensity projected and analysed for particles with a diameter larger than 1 µm and a circularity of 0.5. The threshold criteria were set via ImageJ ‘Robust Automatic Threshold Selection’ (RATS; noise = 11, *λ* = 3, min = 205) [40].

### 2.8. Hyperspectral CARS microscopy

A dual-output laser source (Leukos-CARS; Leukos) was used for the pump and Stokes beams. The source was a passively Q-switched 1064 nm microchip laser, delivering sub-nanosecond pulses at a 32 kHz repetition rate and an average power of approximately 300 mW. For pump (and probe), the fundamental beam at 1064 nm was used, and the Stokes was a fibre-generated super-continuum with a spectral density of more than 100 µW nm^−1^ from 1050 to 1600 nm. The Stokes and pump/probe beams were matched in time and space at the focus of our microscope, as described previously [41]. Briefly, we used a reflective collimator (RC04APC-P01; Thorlabs) to collimate the Stokes beam and filtered it through a longpass 700 nm filter (FEL0700; Thorlabs) and longpass 830 nm filter (LP02-830RS-25; Semrock). The Stokes and pump beams were combined at a dichroic mirror (LP02-1064RU-25; Semrock) and introduced into a modified inverted microscope (Eclipse Ti; Nikon). The pump and Stokes pulses were tightly focused onto the sample with a near-infrared objective (PE IR Plan Apo 100×, NA 0.85; Olympus), resulting in approximately 32.5 mW of total laser power at the sample. The sample was mounted on a stepper-motor stage for coarse positioning (Microstage; Mad City Labs) with a nested piezo stage (Nano-PDQ 375 HS; Mad City Labs) for sample scanning. Together, this provided a 25 mm travel range with sub-nanometre resolution. The CARS signal generated by the sample was collected in the forward direction by another objective (M-10×, NA 0.25; Newport) and sent through notch (NF03-532/1064E-25; Semrock) and short-pass filters (FES1000; Thorlabs) to remove the pump and Stokes radiation. The filtered CARS signal was dispersed by a spectrometer (Shamrock 303i, 300 lines mm^−1^, 1000 nm blaze; Andor) and detected on a deep-depletion CCD (Newton DU920P-BR-DD; Andor). The sample was raster scanned across the laser focus with steps of 0.3 µm in plane. For each position in the sample, a CARS spectrum in the range between 600 and 3400 cm^−1^ was acquired. CARS data were acquired with a pixel dwell time of 100–200 ms, depending on the signal level. The spatial resolution of the instrument was independently measured to be approximately 0.5 × 0.5 × 3.5 µm^3^, and the spectral resolution was limited by the camera pixel pitch to approximately 4 cm^−1^ per pixel. The entire CARS microscope is controlled with custom software written in LabVIEW (National Instruments).

### 2.9. Hyperspectral data analysis

Raw CARS spectra were analysed with custom routines in IgorPro v. 6.37 (Wavemetrics). Initially, data were normalized by exposure time. Treatment of the data with a modified Kramers–Kronig [42] algorithm and error phase correction removes the non-resonant contribution to the signal and retrieves the imaginary component of the third-order Raman susceptibility. For error phase correction, we use an iterative, noise-maintaining approach based on [43] that is modified to be model-free by employing a second-order Savitky–Golay smoothing filter, having a window size of 101 spectral points (404 cm^−1^ width), for repetitive baseline estimation. The window size and order were determined empirically but are the same for all data in this work. These resulting spectra are linear in concentration and can be quantitatively analysed similar to Raman spectra, and we refer to them as Raman-like (RL) spectra [33,44]. Images of particular Raman frequencies were generated in IgorPro for subsequent analysis. Intensity plots of the CH_2_ symmetric vibrations were constructed by integrating the intensity from RL CARS spectra from 2845 to 2852 cm^−1^, and these images were used for identifying oil bodies in all samples imaged with hyperspectral CARS microscopy. Average RL spectra were collected from regions of interest (ROIs) of identified oil bodies and curves were fitted to a sum of Lorentzian peaks for quantitative analysis (electronic supplementary material, figures S5 and S6, tables S1 and S2).

## 3. Results

### 3.1. Spatio-temporal oil body changes during germination

It has been reported that oil bodies undergo morphological changes after imbibition of soya beans, but the dynamics of these changes regarding lipid distribution within the native, germinating cotyledon is not known [45–47]. Therefore, we monitored oil bodies in different parts of the soya bean cotyledon using CARS microscopy, which is a type of CRM. In CARS, multiple excitation lasers are employed: pump, Stokes and probe, to excite Raman vibrational modes in a sample. When the energy difference between the pump and Stokes lasers, Δ_Raman_ = *ω*_pump_ – *ω*_Stokes_, is resonant with a vibrational transition of bonds in the focal volume of the lasers, the CARS signal is strongly enhanced, providing a chemically specific, label-free signal suitable for *in situ* imaging. By tuning Δ_Raman_ to 2845 cm^−1^, the resonance frequency for CH_2_ (methylene) groups in lipids, we imaged the oil bodies in living and fixed cotyledons with three-dimensional spatial resolution similar to multi-photon fluorescence microscopy without the need for exogenous labels [48].

**Figure 1.**
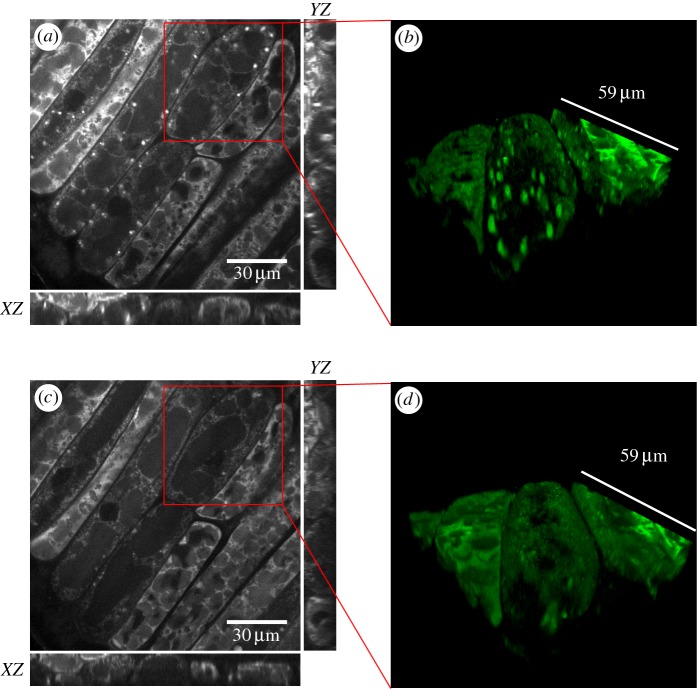
CARS image plots of a living, cut-open soya bean using the CH_2_ symmetric stretch vibration (2845 cm^−1^) intensity as contrast, at (*a*) day 4 and (*c*) day 5 post imbibition. Large oil bodies are depleted in most of the depicted cells by day 5. (*b*,*d*) Three-dimensional reconstructions of the regions highlighted in (*a*) and (*c*).

Because sample perturbation by the excitation light is minimal (as explained below), CARS microscopy allows for three-dimensional time lapse imaging in living, germinating soya beans. In figure 1, we show still images from a 24 h timelapse video starting at day 4 (post imbibition) from the adaxial (inner) region of the cotyledon. The orthogonal views as well as the three-dimensional reconstruction illustrate that the penetration depth for imaging is sufficient to span at least one cellular layer into a living cotyledon. From day 3 to day 5, we observed a quadruplication of root length (electronic supplementary material, figure S1), and the live imaging movie shows highly mobile lipids in living cells (electronic supplementary material, movie S1) over the same period. The live CARS imaging of lipid distribution in soya bean cotyledons shows that oil body size and distribution are highly dynamic during germination. Importantly, we note that the imaged cotyledon developed into a full plant within 8 days (electronic supplementary material, figure S3), which proves that extended CARS imaging was not cytotoxic.

**Figure 2.**
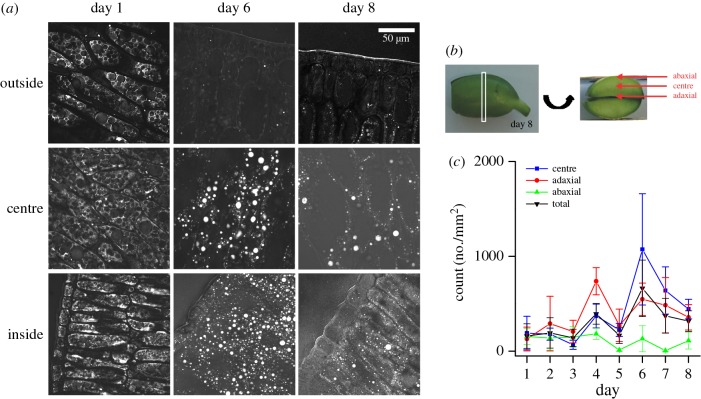
(*a*) Representative CARS images obtained using the CH_2_ symmetric stretch vibration (2845 cm^−1^) of lipids as contrast, at days 1, 6 and 8 of soya bean cotyledons post imbibition. Samples were imaged after fixation and cryo-slicing (15 µm thickness). Images show location-dependent oil body size changes and the disappearance of lipids from the abaxial region of the cotyledon. (*b*) Schematic showing the regions of the soya bean cotyledon for image acquisition. All images were taken 3–5 mm from the embryo. (*c*) Quantification of oil body density for large oil bodies: diameter more than 3 µm, using the RATS thresholding on maximum intensity projected images from six image sub-stacks as described in the Material and methods. Quantification of large oil bodies showed the appearance of larger entities at day 4 and day 6, for the adaxial and centre, respectively. Errors bars are standard deviation from quantification in three separate sections.

Upon examination of three spatial regions within fixed and sectioned cotyledons: the (flat) adaxial, centre and (curved) abaxial (outer) regions over days 1–8 of germination, we clearly see a depletion of lipids from abaxial cell layers and an increase in lipid signal in the adaxial and central regions (figure 2*a*). Large round features appear in the centre and adaxial regions by day 6. CARS images reveal that oil bodies in different regions of the cotyledon undergo unique morphological changes throughout germination. Considering that different regions of the cotyledon become different parts of a plant, this is not unexpected. The adaxial region differentiates into the typical morphology of a leaf-like mesophyll by day 8 [49]. All cells in the abaxial cotyledon will eventually become a (woody) epidermis, which should be void of organelles, leaving behind virtually no lipid-containing structures. This is consistent with the CARS images in figure 2, which show that the abaxial region is devoid of lipid signal by day 6. Interestingly, the images of the adaxial and centre regions of the cotyledon show an increase in oil body size at day 6, followed by a decrease at day 8.

Quantification of the number of CH_2_-rich oil bodies larger than 3 µm in diameter (defined as ‘large’ oil bodies) from the three different cotyledon regions shows the spatio-temporal behaviour of oil body morphology during germination (figure 2*c*). Growth and subsequent shrinkage of oil bodies was most apparent within the centre region, and to a lesser extent in the adaxial region, with the centre region having a slightly delayed oil body size increase compared with the adaxial region. The abaxial region of the cotyledon showed a marginal oil body size increase but significantly less than either the adaxial and centre regions. In addition to oil body size quantification from CARS, oil body size was measured using laser diffraction measurements on extracted oil bodies from day 1 to day 8 germinating plants. These results, which are binned over the entire germinating soya bean cotyledon, independently confirm that oil body size increases, then decreases and is consistent with the quantification of large oil bodies from CARS (electronic supplementary material, figure S4).

### 3.2 Oil body lipid composition undergoes subtle changes while protein emulsifier disappears during germination

With multiple experimental methods confirming oil body morphological changes during germination, we next investigated whether the biochemistry of oil bodies changed during germination. We used hyperspectral CARS imaging—where a full vibrational spectrum at each spatial location is acquired—to measure oil body composition *in situ* in fixed cotyledon sections. After appropriate processing, these spectra provide a directly quantifiable vibrational RL spectrum, depicting local chemistry within each spatial voxel (0.5 × 0.5 × 3.5 µm^3^) [50]. Hyperspectral datasets were obtained from days 1, 5 and 9 of germinating soya bean cotyeldons from the central region.

**Figure 3.**
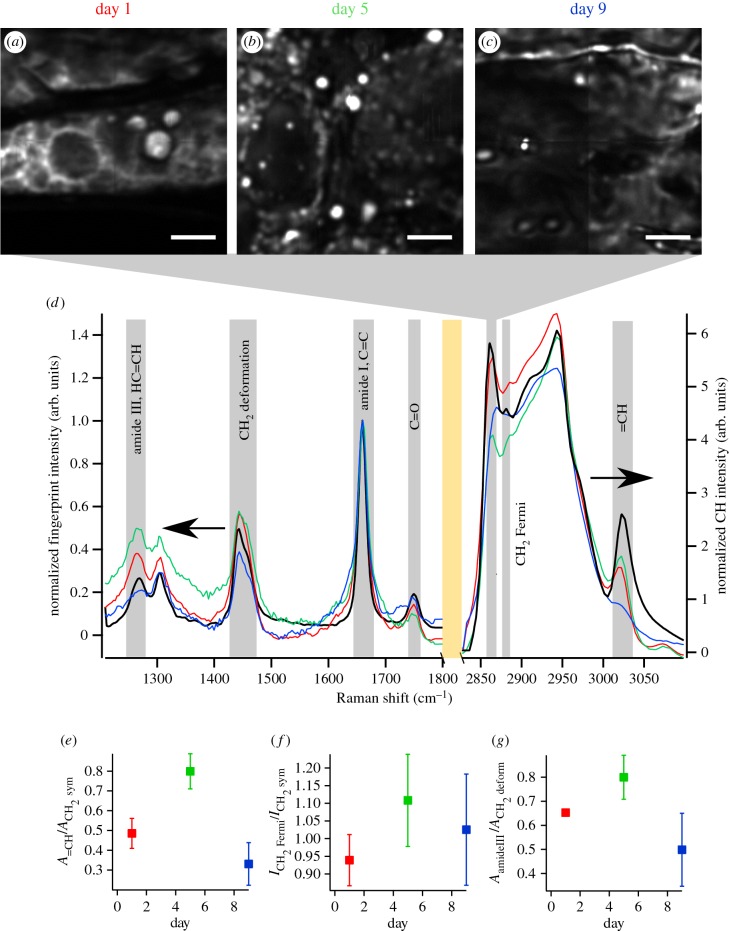
Plots of the CH_2_ symmetric stretch intensities (integrated over 2845–2852 cm^−1^ of RL spectra) from hyperspectral CARS microscopy datasets of soya bean cotyledon sections at (*a*) day 1, (*b*) day 5 and (*c*) day 9 post imbibition. (*d*) Average, normalized RL spectra from at least four different CH_2_-rich ROIs in the respective images show the local composition of oil bodies with same colour scheme as in (*a*). The spectrum of refined soya bean oil is shown in black. Grey rectangles show specific vibrational bands, and the orange rectangle marks the division between the fingerprint to CH regions of the graph. (*e*) Lipid unsaturation, as indicated by 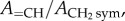 where *A* is the peak area determined from multi-peak fitting, increased from day 1 to day 5, and dropped below the starting value at day 9. (*f*) Lipid chain order 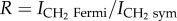, where 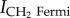 and 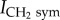 are the RL spectral intensities from 2885 and 2848 cm^−1^, respectively, increased from approximately 0.94 at day 1 to 1.13 at day 5 before decreasing again to 1.02 by day 9. (*g*) Protein content, as assessed by 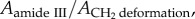 dropped at day 9 following an increase at day 5 when compared with day 1. Error bars in (*e–g*) are standard deviation calculated from *N* > 4 spectra from ROIs within each sample. ROIs always consisted of at least 10 voxels (spectra) for averaging. Scale bar in (*a*–*c*) is 10 µm.

Oil bodies in hyperspectral CARS images were identified from images rendered by plotting the CH_2_ (2845–2852 cm^−1^) intensities that are characteristic of aliphatic carbons (figure 3*a*–*c*). This is a similar contrast metric to that used to produce figures 1 and 2. We note that, in contrast to the semi-quantitative data in figures 1 and 2, the data in figure 3*a*–*c* are amenable to quantitative band analysis [50]. To quantitatively examine oil body-specific chemistry during germination, spectra from at least four CH_2_-rich ROIs for each time point were averaged; these are shown in figure 3*d*. In order to better compare differences in RL spectra from each day, we plot the two regions of the spectra as normalized spectra in figure 3*d*. For reference, an RL spectrum of refined, analytical soya bean oil (analytical standard from Sigma) is also shown in figure 3*d* (black curve). The ‘fingerprint’ region (900–1800 cm^−1^) and ‘CH’ region (2700–3100 cm^−1^) were normalized by the amide I/C=C vibration (1646 cm^−1^). Changes in local lipid composition were further quantified by analysing the ratios of specific vibrational resonances in the spectra, which correspond to different functional groups. This was done by multi-peak fitting the fingerprint and CH spectra from at least four different ROIs from each day with sums of Lorentzian functions corresponding to specific vibrations with centre and width parameters found in the established literature [51–53]. Results from the fitting are shown in the electronic supplementary material, figures S5 and S6, and were in good agreement with the RL spectra from each day, and parameters of each Lorentzian as well as the specific vibration are given in the electronic supplementary material, tables S1 and S2.

The peak at approximately 3005 cm^−1^ originates from =CH vibrations from unsaturated carbons in acyl chains. Therefore, the ratio of peak areas 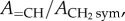 where *A* is the peak area obtained from multi-peak fitting, is a metric for the relative degree of lipid unsaturation [44,54]. The observed level of unsaturation was markedly increased at day 5 after imbibition (0.80 ± 0.09) (mean ± s.d.) compared with day 1 (0.49 ± 0.08) and dropped below the starting value by day 9 (0.33 ± 0.11) (figure 3*e*).

Beyond relative unsaturation, our data permitted calculation of the molar average lipid chain length and number of unsaturated (C=C) bonds. This was done by measuring the RL spectrum of a series of TAGs as well as a protein solution and using these spectra to construct a series of component spectra via least-squares decomposition that represent the TAG background, TAG number of C=C bonds, TAG number of CH_2_ groups, and protein (see the electronic supplementary material, Methods). These component spectra are shown in the electronic supplementary material, figure S7. To validate this method, we calculated the average chain length and number of C=C bonds of analytical standard olive, sunflower, canola, soya bean and peanut oils as well two TAG mixtures by using non-negative least-squares fitting of measured spectra with derived component spectra (see the electronic supplementary material, Methods, equation S1). This process is related to partial least-squares decomposition, which has been extensively used for characterization of fatty acids and proteins in a variety of plant and food sources with near-infrared spectroscopy [55–57].

The fits with the derived component spectra model match well with the measured spectra (electronic supplementary material, figure S7), and produced average chain lengths and numbers of C=C bonds that were accurate to within 0.5 C and 0.1 C=C bonds, respectively, when compared with molar averages of analytical standard oils from manufacturers' provided mass spectrometry data (electronic supplementary material, table S3). We found that the average lipid chain was 17.9 C with 1.1 C=C per chain at day 1; 18.0 C and 1.4 C=C per chain at day 5; and 14.4 C and 0.5 C=C per chain at day 9 when fitting the spectra shown in figure 3*d*. These numbers are summarized in table 1.

**Table 1.** Chain length, number of C=C bonds, and protein : lipid ratio for spectra in figure 3*d* determined using the method described in the text and electronic supplementary material, Methods. Error was determined by RMS deviation from gas chromatography molar averages of analytical standard oils (electronic supplementary material, table S3). The protein : lipid ratio is a relative metric for comparison among samples but is not an absolute, stand-alone metric.

	molar average chain length	molar average number of C=C bonds	protein : TAG ratio (unscaled)
day 1	17.9 ± 0.5	1.1 ± 0.1	0.17 : 1
day 5	18.0 ± 0.5	1.4 ± 0.1	0.38 : 1
day 9	14.4 ± 0.5	0.5 ± 0.1	0.27 : 1

The increase in unsaturation at day 5 observed from our chain length calculation is in line with the observation of increased lipid unsaturation calculated from the spectral ratios (figure 3*e*). In addition to chain length and unsaturation, we quantified changes in lipid order in the oil bodies. The ratio of peak intensity of the CH_2_ Fermi resonance (approx. 2885 cm^−1^) to CH_2_ symmetric (approx. 2845 cm^−1^) is related to the ‘order’ or packing of aliphatic side chains [58,59]. During germination, the order parameter, 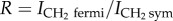, where *I* is the intensity of the RL spectra at 2885 cm^−1^ (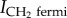) and 2845 cm^−1^ (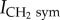), increased from 0.94 ± 0.07 at day 1 to 1.13 ± 0.13 at day 5 and decreased to 1.02 ± 0.16 at day 9 (figure 3*f*).

*In situ* spectroscopy data also allowed for determination of the relative amount of protein associated with oil bodies and how it changed during germination. The amide III vibration (1252 cm^−1^) is a proxy for protein concentration, and a decrease in the amide III band is clear, especially at day 9. The ratio of peak areas, 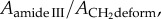 was then used as a metric of protein-to-lipid ratio associated with oil bodies. 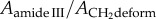 showed a decrease to 0.5 ± 0.15 at day 9 (figure 3*g*) after a slight increase from day 1 to day 5 (0.65 ± 0.01 to 0.8 ± 0.09). This is consistent with the trend in protein : TAG ratio obtained from spectral fitting for our chain length calculations (table 1). We found that the protein : TAG ratio is 0.17 at day 1, 0.38 at day 5, and 0.27 at day 9. In support of these quantitative metrics, we generated images of protein distribution via the ring breathing mode (approx. 1003 cm^−1^) and compared them with images of the CH_2_ symmetric vibration (approx. 2845 cm^−1^) at days 1, 5 and 9 of germination (electronic supplementary material, figure S9). While it was not clear from these images if a quantitative increase or decrease in oil body-associated proteins occurred during germination, it was certainly clear that oil bodies in cotyledons were always surrounded by a substantial pool of proteins and that less lipid–protein colocalization (yellow in the electronic supplementary material, figure S9) appears at day 9 than at day 1 or day 5. Finally, from both hyperspectral CARS quantification and imaging, we note that oil bodies became more heterogeneous with increasing germination time. This is reflected by the increased spread of the quantities reported in figure 3*e*–*g* (i.e. the error bars increase with time) as well as the less structured images in the electronic supplementary material, figure S9.

**Figure 4.**
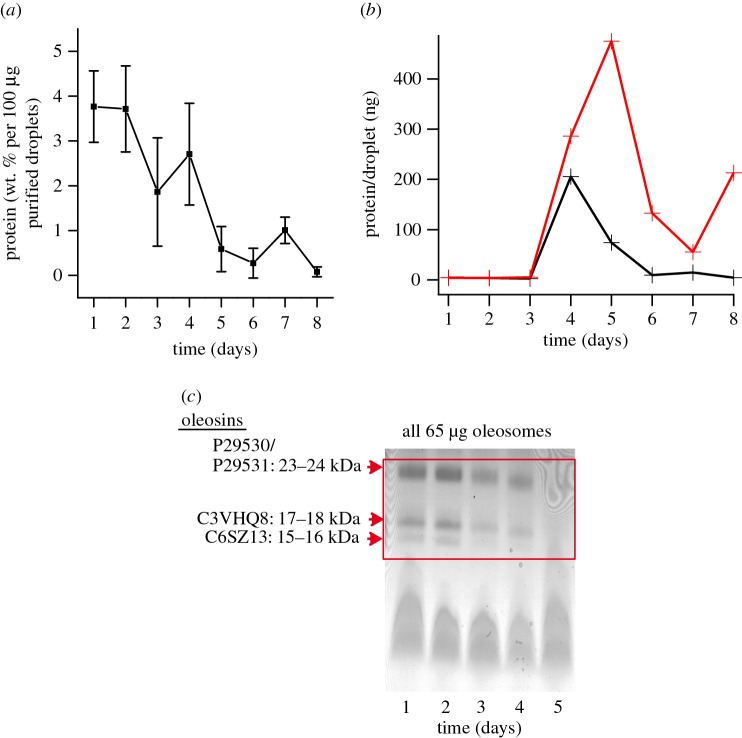
(*a*) Bradford assay to quantify weight % of total protein associated with oil bodies. All samples had the same dry weight of oil bodies. Error bars are standard deviation from the three independent oil body extractions. (*b*) Calculation of protein per oil body, based on the size of extracted oil bodies (electronic supplementary material, figure S4) and Bradford measurements with 100 µg of purified oil bodies. A constant density of oil (gm ml^−1^) was assumed for the oil bodies at each day. The red line shows the calculated protein per oil body by keeping weight % of protein constant at the Bradford measurement from day 1 and varying only the number of oil bodies according to the change in size (electronic supplementary material, figure S4). The black line is the measured protein per oil body calculated using the actual Bradford measurement at each day along with the change in oil body size. The difference between the black and red lines indicates that the amount of protein associated with oil bodies was actively changed during germination. (*c*) SDS–PAGE showing reduction of oleosins during germination. Oleosins are no longer visible after day 5 via Coomassie staining.

Since hyperspectral CARS analysis of the oil bodies at days 1, 5 and 9 showed that protein concentration associated with oil bodies changes throughout germination, we further analysed protein content, and specifically oleosin emulsifiers, associated with extracted oil bodies. At day 1, we observed that 3.8 weight % of the extracted oil weight was protein, and this decreased steadily to 0% by day 8 as measured by a Bradford assay (figure 4*a*). From laser diffraction experiments, where we observed the average diameter of oil bodies at each day of germination (electronic supplementary material, figure S4), and the Bradford measurements, we determined the average protein content per oil body at each day. This was done by calculating the number of oil bodies analysed in the Bradford measurement, assuming (i) a spherical shape for oil bodies with diameter as determined for the electronic supplementary material, figure S4, and (ii) a constant oil density (0.917 gm ml^−1^ at 25°C) throughout germination. We found that protein content per oil body increases sharply at day 4 and rapidly decreases again before reaching zero at day 8 (figure 4*b*, black). When we compare the protein per oil body from the Bradford measurements with the protein per oil body expected from oil body size changes alone—i.e. a constant Bradford signal at each day—(figure 4*b*, red), it is clear that changes in oil body size alone cannot explain the experimentally determined protein density. The measured protein per oil body is consistently lower from day 2 onward. Interestingly, the increase (from day 1 to day 4) and decrease (from day 4 to day 8) in measured protein density in figure 4*b* is similar to the trend seen in figure 3*g* (and table 1), where the protein per lipid increases at day 5 relative to day 1 and then decreases at day 9.

Lastly, we independently verified that oleosins associated with oil bodies decreased over the course of germination using SDS–PAGE of purified oil bodies. High (23–24 kDa) and low (15–16 and 17–18 kDa) molecular weight soya bean oleosins were visible starting from day 1 but decreased until they were undetectable at day 5 (figure 4*c*). Similar degradation of oleosins was previously reported [16,60] in soya beans. This verifies that oil bodies start as oleosin-coated oil bodies to become non-oleosin-coated oil bodies during the early days of germination [34].

## 4. Discussion

In this work, we monitored oil body dynamics, quantified oil body morphology and quantified changes in oil body chemistry during germination with label-free CARS microscopy *in situ* via direct visualization using the vibrational modes of methylene groups (lipids) in living, intact tissues. Monitoring lipid morphology with CARS was similar to what was previously done by Cavonious *et al.* [61]. In that study, changes in oil body size in algae were measured with CARS and correlated with mass spectrometry in control and nitrogen-starved conditions, whereas the current study focused on oil body changes in unperturbed germination. Our results show that oil bodies first grow in size, followed by shrinkage, and these processes occur at different rates, depending on the location within the cotyledon. The abaxial region of the cotyledon empties of all lipids by day 5, while the centre and adaxial regions of the cotyledon show a time-dependent size of oil bodies. The spatially specific oil body dynamics are analogous to the unique spatio-chemical composition of oil bodies in cotton shown by MALDI-IMS imaging [25,26]. In that work, the different locations within the seed showed distinct lipid compositions, and these regions were known to develop into different plant organs/tissues. This underscores the simultaneous heterogeneous metabolic processes during early germination at different locations within a cotyledon. The observed morphological changes of oil bodies were paralleled by changes in their local lipid composition and associated protein composition. We note that our measurements of oil body size and chemistry changes do not distinguish between *de novo* synthesis of oil bodies and alteration of existing oil bodies. While it is likely that new oil body synthesis is occurring—diacylglycerol acyltransferase is surprisingly active in *Arabidopsis* germinating seeds and seedlings and has also been found in germinating soya beans—it is typically assumed that germinating soya beans primarily consume existing oil bodies imparted during seed maturation [3,62–64]. This is especially true for etiolated soya bean germination (in our case, days 1–5) [63]. Furthermore, TAG content has been shown to clearly decrease throughout soya bean germination [64,65]. Nevertheless, we are unable to rule out the possibility that changes in oil body synthesis during germination, in addition to consumption and metabolism, influence the results presented here.

A pattern of growth and shrinkage of oil bodies in early plant growth was very recently identified in native *Arabidopsis* embryos using confocal fluorescence microscopy [6]. Our results are generally consistent with that study, even though the plant orders (*Brassicales* versus *Fabales*), spatial location in the plant (embryo versus cotyledon) and developmental stage (post germination versus imbibition) of the samples are different. Moreover, our results showing more lipid content in the inner regions of the cotyledon are also generally consistent with those from NMR imaging by Borisjuk *et al*. [29] in whole soya bean (*Glycine max*) cotyledons; however, the exact timing (particular day) within germination of the NMR images was not stated.

Quantitative hyperspectral CARS imaging showed that oil bodies exhibit a higher degree of lipid unsaturation at day 5 compared with just after imbibition (day 1) or towards the end of germination (day 9). This is in line with previous studies of germinating seeds, where stearic acid and palmitic acid were reported to be replaced by linolenic acid in germinating sunflower seeds [66]. Such a transformation indicates that saturated lipids are metabolized first following imbibition. The increase in unsaturation we observe was accompanied by a slight increase in aliphatic chain order *R*, which correlates with the degree of packing of lipid side chains, in oil bodies. This counterintuitive result is possibly related to the increase in protein content associated with droplets at day 5; however, further work is warranted to clarify this issue.

Our data show that the average chain length of lipids in oil bodies approximated that of refined soya bean oil (17.5 C from our measurements) at day 1 (17.9 C) and day 5 (18 C). The number of C=C bonds per chain increased at day 5 (to 1.4 C=C bonds), and both the calculated average lipid chain length and unsaturation very closely matched that of refined soya bean oil (1.6 C=C bonds). At day 9, both the chain length and unsaturation of the lipids in oil bodies decreased, perhaps indicative of consumption of deposited oil body lipids during seed maturation, and more representative of *de novo* synthesized oil bodies. From the perspective that oil consumption (as opposed to biogenesis) dominates lipid turnover during early germination (day 1 to day 5), this suggests that longer chain lipids are metabolized first, perhaps because more metabolites (acetyl-CoA) in glyoxysomal β-oxidation are derived from longer chain lipids than from lipids with shorter chains. In addition, the use of 16 or 18 C lipid chains in membranes for organelle development could also explain why shorter chains remain at day 9 [67]. The increased unsaturation seen at day 5 is consistent with the work from Yoshida [65], who showed that TAGs containing saturated fatty acids were hydrolysed earlier in germination than unsaturated fatty acids. Finally, we note that germinating soya beans were exposed to light on day 5, so photosynthesis was in strong effect by day 9, as evidenced by the strong green colour. This may have caused changes in lipids stored in oil bodies, e.g. for production of lipids (thylakoid membranes) that support photosynthesis. Clarifying the complete lipid biochemical changes in soya bean oil bodies throughout germination requires further investigation, potentially via MALDI-ISM, to elucidate the dynamics of different lipid subtypes within oil bodies in soya bean cotyledons.

We further explored the protein biochemistry of the oil bodies throughout germination and found a decrease in proteins associated with oil bodies in hyperspectral images (figure 3*g* and electronic supplementary material, figure S9) and bulk biochemical analysis (figure 4*b*). It is clear from the electronic supplementary material, figure S9, that protein-rich regions and oil bodies appeared to colocalize (to within 350 nm) at days 1 and 5, but this colocalization was less apparent by day 9, which qualitatively agrees with the quantitative data from spectral analysis. Oil body-bound oleosins completely disappeared by day 6, as seen from the SDS–PAGE analysis. Decreased presence of proteins, and specifically oleosins (which are very strong emulsifiers because of their strong negative charge and large steric hindrance), consigns only the phospholipid monolayer to stabilize the oil bodies. This will weaken the emulsification system and promote oil body coalescence, consistent with the oil body size increase observed at day 5 (figure 2 and electronic supplementary material, figure S4). Interestingly, we observed that oil bodies decreased in size (days 6–8) after initially increasing. This is correlated with maximal lipolytic activity in glyoxysomes [68,69] and β-oxidation of fatty acids [70] as well as oxygenation of polyunsaturated activity by lipoxygenases [69,71], suggesting a possible connection between enzymatic hydrolysis of TAGs, oleosin bound to the oil body and lipid metabolism. This conjecture is further supported by the close proximity of glyoxysomes to oil bodies in soya beans that has been observed with electron microscopy [72,73].

Finally, it is interesting to note that soya beans are known to have especially small oil bodies immediately after imbibition. Comparing oil body remodelling in different plants that have larger initial oil bodies using *in situ* CARS microscopy might help elucidate the underlying physiological reasons for the morphological changes in oil body size and composition during plant development. Furthermore, combining the CARS label-free approach for lipid (and protein) imaging with organelle-specific stains for mitochondria or glyoxysomes will be a useful platform to explore how oil body storage and metabolism are related in different parts of cotyledons during germination.

## 5. Conclusion

In this work, we used label-free CARS microscopy to observe intracellular morphological and chemical changes in oil bodies in germinating soya bean cotyledons *in situ*. We showed that CARS imaging over a 24 h period did not arrest cotyledon development and that CARS is a convenient, label-free method to analyse spatio-temporal oil body chemistry as it does not rely on dye permeability or intricate sample preparation. Based on *in situ* chemical imaging with hyperspectral CARS, we found marked variations in the lipid composition within, and protein associated with, oil bodies throughout the germination process. Bulk biochemical analyses of parallel samples used for CARS imaging showed that proteins associated with oil bodies, and especially oleosin emulsifier proteins, were reduced over the first 6 days of germination, coincident with oil body size increase. The spatio-chemical changes in oil bodies accessible with label-free CARS microscopy opens new possibilities for studying lipid dynamics and flux of lipids from oil bodies into other organelles during plant development.

## Supplementary Material

Supplementary Information

## Data accessibility

For numerical data in the main text of this manuscript, see [74]. The image data and supplementary numerical data are available from the corresponding author.
