# Supplementary material for: Label-free in situ imaging of oil body dynamics and chemistry in germination
Source: J R Soc Interface. 2016 Oct;13(123):20160677. doi: 10.1098/rsif.2016.0677 (PMC5095225; doi:10.1098/rsif.2016.0677)
Supplement: Supplementary Information [file rsif20160677supp1.pdf]

Supplementary Information

Label-free in situ imaging of oil body dynamics and chemistry in germination

Gustav Waschatko[1,#], Nils Billecke[1,#], Sascha Schwendy[1], Henriette Jaurich[2], Mischa Bonn[1], Thomas A. Vilgis[2], Sapun H. Parekh[1,*]

[1] Department of Molecular Spectroscopy, Max Planck Institute for Polymer Research, Mainz, Germany

[2] Department of Polymer Theory, Max Planck Institute for Polymer Research, Mainz, Germany

[#] These authors contributed equally to this work

[*] Correspondence should be sent to: parekh@mpip-mainz.mpg.de

Supplementary Methods

Laser diffractometry

The size distribution of extracted oil droplets at different germination stages was measured after 3-fold dilution of the extracted oil droplets in phosphate-buffered saline (PBS) using an LS 13 320 (Beckman Coulter, Brea, USA) laser diffraction analyzer. Care was taken to measure the size distribution directly after resuspension in PBS to prevent further coalescence. Three samples were measured from each droplet extraction, and three extractions (growth cycles) were conducted for each specified germination day. All data for a given extraction (and day) was pooled and fit using size models provided by the manufacturer software. The Beckman Coulter LS 13 320 software (version 6.1) had internal presets for corrections associated with refractive index mismatch between the carrier fluid of 1.332 (PBS/water) and the sample 1.60 (real part) and 0.01 (imaginary part).

Determination of average lipid chain length and number of C=C bonds

All materials, unless otherwise stated, were purchased from Sigma, and part numbers are given in parentheses. Pure TAG oils: 8:0 (T9126), 16:1 (T2630), 18:1 (T7140), 18:2 (T9517), and 18:3 (T6513), a 50%-50% mixture (v/v) of 18:2 and 8:0, and Bovine Serum Albumin (BSA) (05470) were measured with hyperspectral CARS as a basis set for least squares decomposition. TAG oils were measured as-received whereas BSA was diluted to 100 mg/mL in Milli-Q water. In addition to TAG oils, analytical standard oils: canola (46961), olive (47118), palm (46962), peanut (47119), soybean (47122), and sunflower (47123) were measured with hyperspectral CARS as well. Each hyperspectral dataset was converted into linear Raman-like data using the exact same algorithm and error phase subtraction as stated in the main text. Similar to data in the main text, the data was cropped to include 990 – 1786 cm^{-1} and 2828 – 3100 cm^{-1}. In addition, all TAGs, TAG mixture, BSA, analytical oils, and soybean spectra were aligned so that the CH_2 deformation peak (nominally at 1440 cm^{-1}) always occurred at 1440 cm^{-1}. Spectra were then normalized to this vibration such that the intensity was 1.

We used a least square decomposition with known covariances in order to construct component spectra for the variables representing the features of interest. The following covariances were used with for basis set spectra.

Molecule	TAG background	#C=C bonds (per chain)	$\#CH_2$ groups (per chain)	Protein
**16:1 TAG**	1	1	12	0
**18:1 TAG**	1	1	14	0
**18:2 TAG**	1	2	12	0
**18:3 TAG**	1	3	10	0
**8:0 TAG**	1	0	6	0
**50% 18:1 TAG + 50% 8:0 TAG (v/v)**	1	0.68	8.05	0
**BSA**	0	0	0	1

Least squares decomposition with these covariances and the measured basis spectra was solved, providing component spectra for TAG background, TAG #C=C bonds (per chain), TAG $\#CH_2$ groups (per chain), and protein. These component spectra were cropped to include a smaller region of the spectrum: from 1200 – 1780 cm^{-1} and 2828 – 3102 cm^{-1}, interpolated by a factor of 6, and used to decompose all measured spectra of the analytical oils (and soybean tissues) – which were also cropped to the same range and interpolated – with the following model:

$$Meas(f) = a * C_1(f - f_0) + b * C_2(f - f_0) + c * C_3(f - f_0) + d * C_4(f - f_1) + m * f + e \quad \text{eq. 1}$$

where:

1. C_1, C_2, C_3, and C_4 are the derived spectral components for TAG background, #C=C bonds per chain, $\#CH_2$ groups per chain, and protein, each having weights *a*, *b*, *c*, and *d*, respectively.
2. *f* is the Raman shift , and f_0 and f_1 are parameters to allow for slight shifts of the component spectra compared to the measured data (*Meas*). The shift values were always less than 4 cm^{-1}.
3. *m* and *e* are parameters allowing a linear baseline, which was sometimes present in the tissue spectra.

When fitting to all analytical standard oils and TAG mixtures, *d* and f_1 were fixed to 0, while all parameters were fit for tissue samples.

In order to produce a chain length and # of C=C bonds, all fit parameters were normalized by *a* in order to make the TAG background (component 1) = 1. This allowed direct extraction the # of C=C bonds as *b/a*, and the chain length = 2* (*b/a*) + *c/a* + 2 (1 each for the methyl and carbonyl end groups).

The components, fits to all analytical oils, and sample spectra are show in Figures S7 and S8.

Supplementary Figures

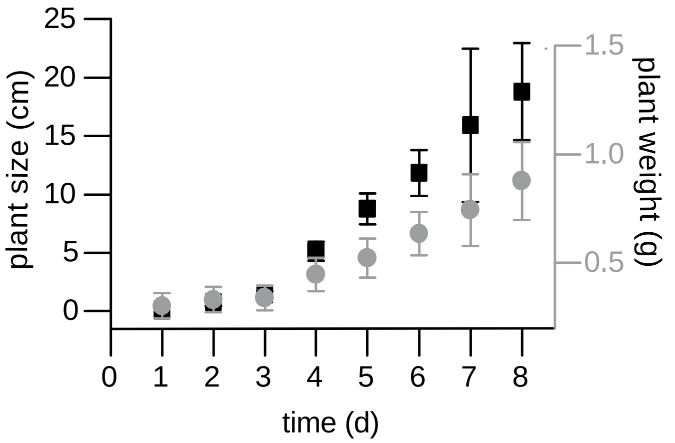

Figure S1. Soybean size and weight at the indicated day post imbibition.

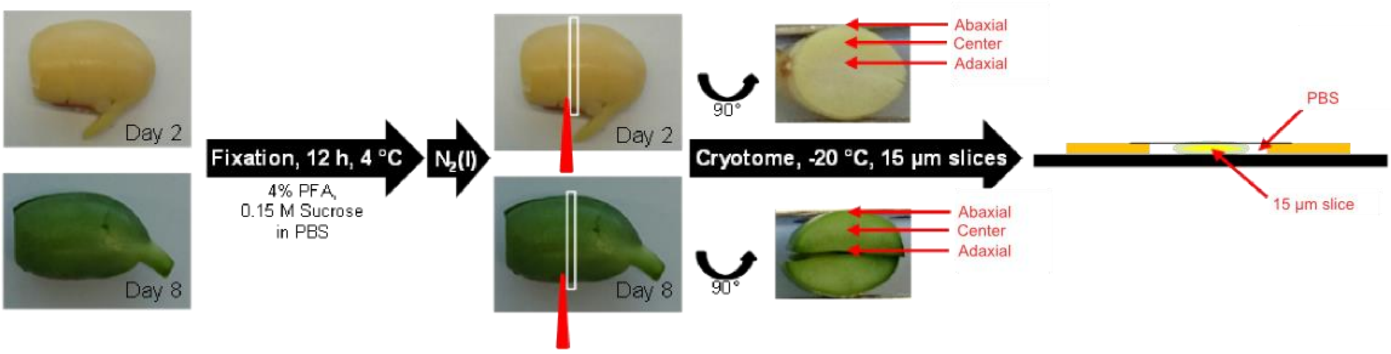

Figure S2. Preparation of paraformaldehyde (PFA) fixed germinating soybean cotyledons for CARS imaging.

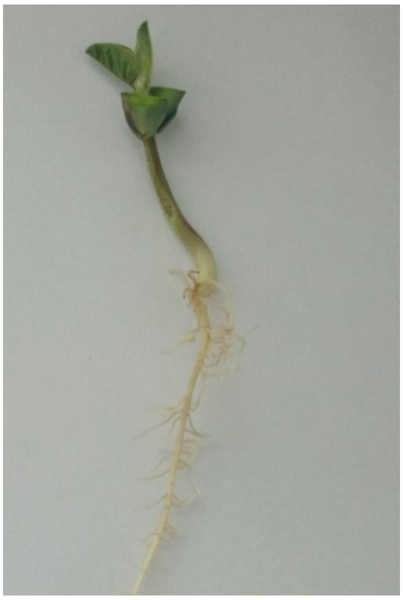

Figure S3. Soybean seedling used for live CARS imaging (Fig. 1 main text) after 8 days of germination.

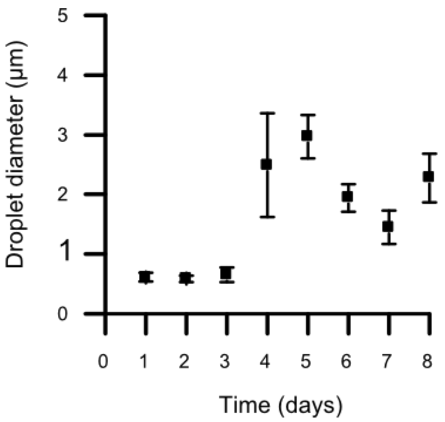

Figure S4. Laser diffraction measurements of extracted oil droplet size at the indicated day post imbibition. Error bars are standard deviation from N = 3 independent growth cycles and extractions.

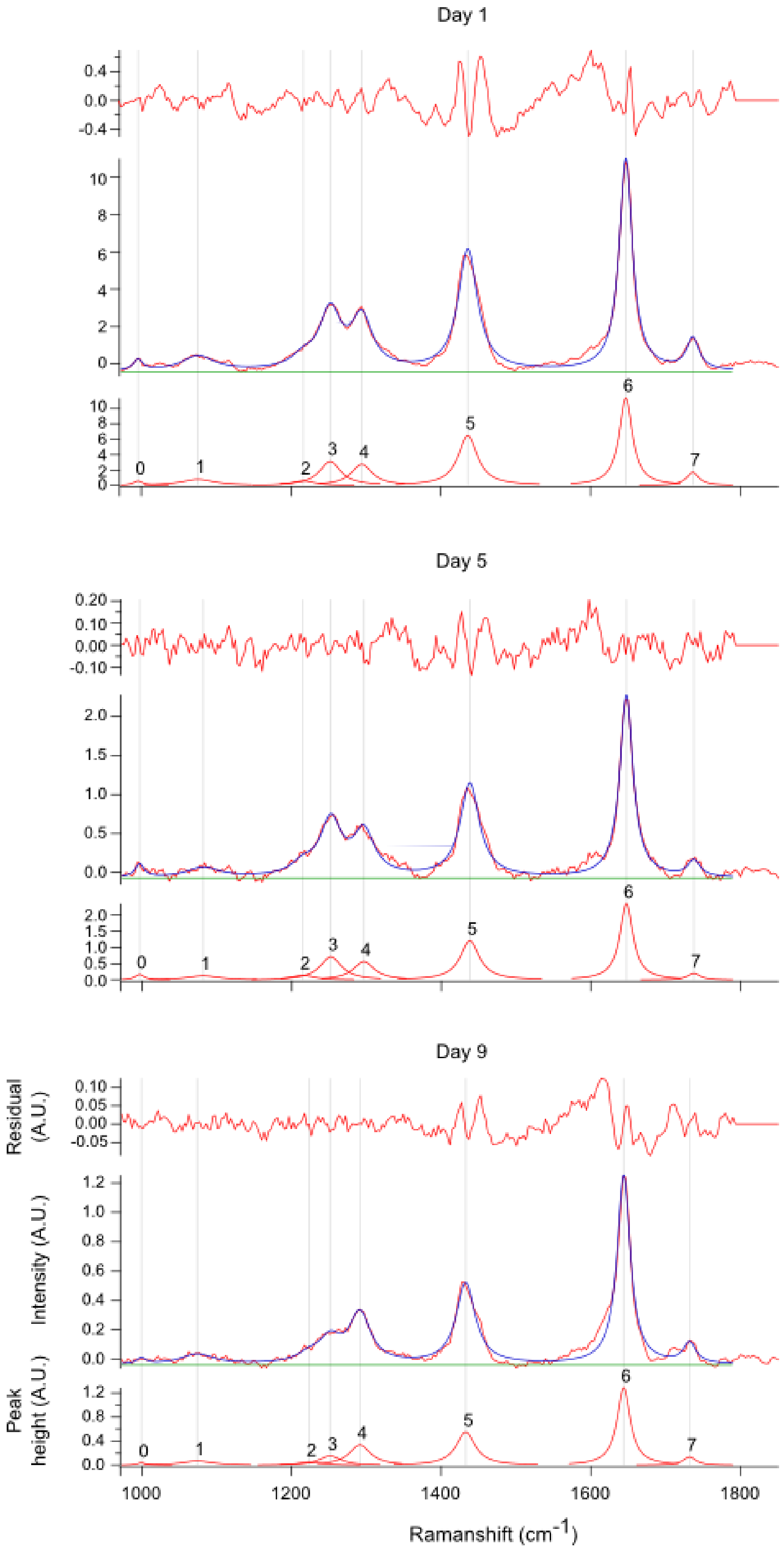

Figure S5. Multi-peak fitting for the Fingerprint region Raman-like spectra from the indicated day post imbibition. The full-width, half-maximum (FWHM) of each peak was held constant for fitting each of the three spectra. Fits are shown in blue. Additional peak fitting parameters are given in Table S1.

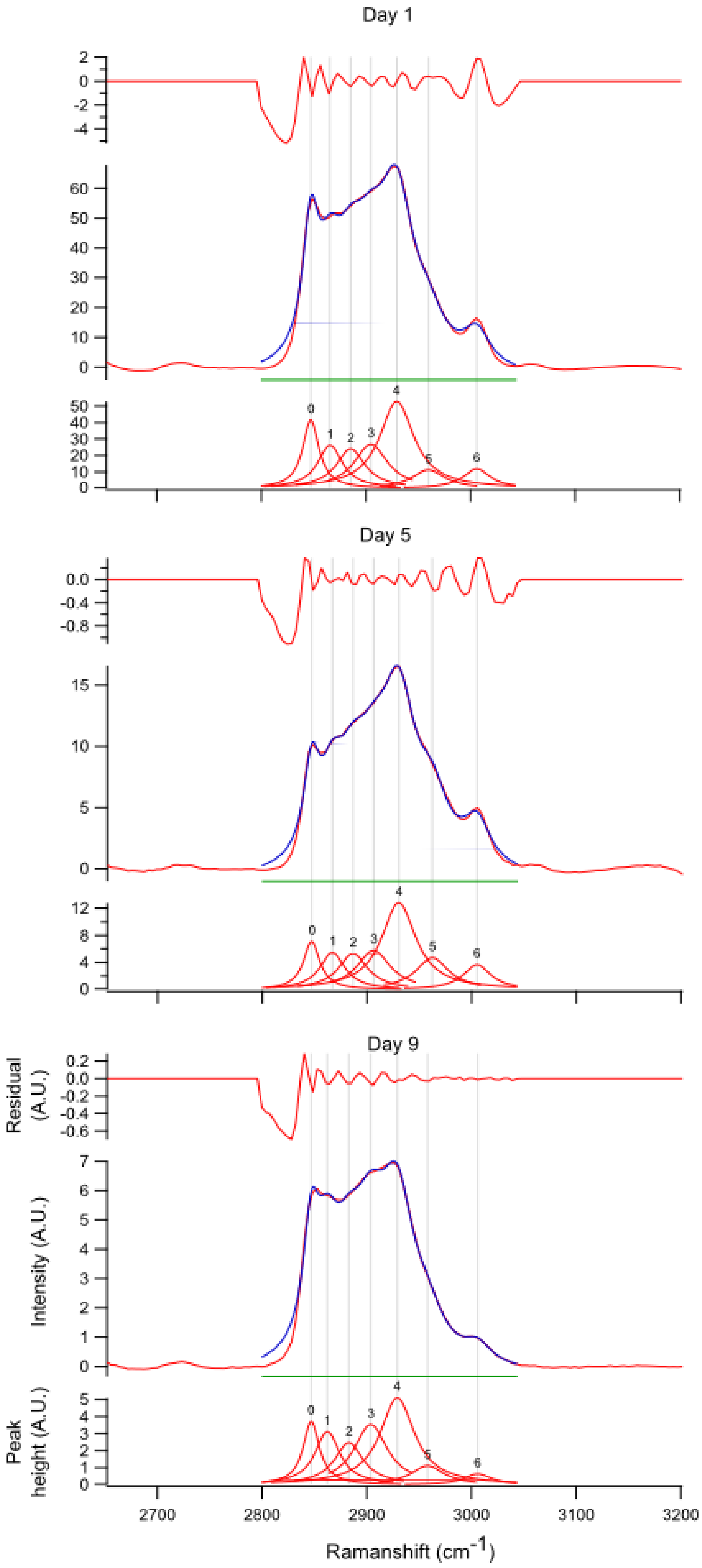

Figure S6. Multi-peak fitting for the CH region Raman-like spectra from the indicated day post imbibition. The FWHM was held constant for fitting each of the three spectra. Fits are shown in blue. Additional peak fitting parameters are given in Table S2.

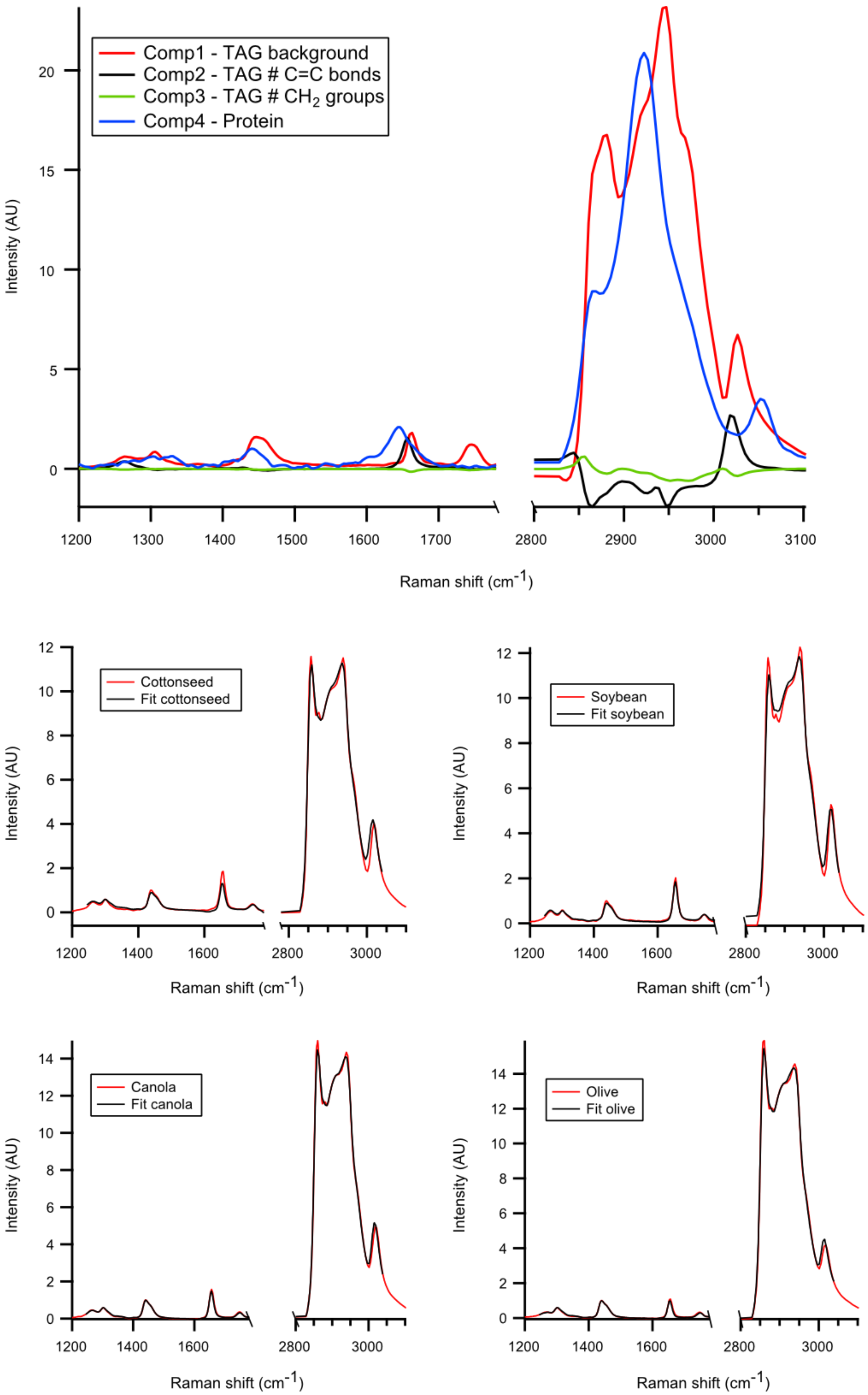

Figure S7. Raman-like spectral components (top) and reconstructed spectra (bottom) for different analytical grade oils. Spectral components were generated by least square decomposition with known covariances to TAG and BSA

spectra. The covariance table is shown in Supplementary Methods. Fits to measured analytical oil spectra were constructed using a linear combination of the components with a linear baseline according to eq. 1. The derived chain length and # C=C bonds from each spectrum are shown in Table S3.

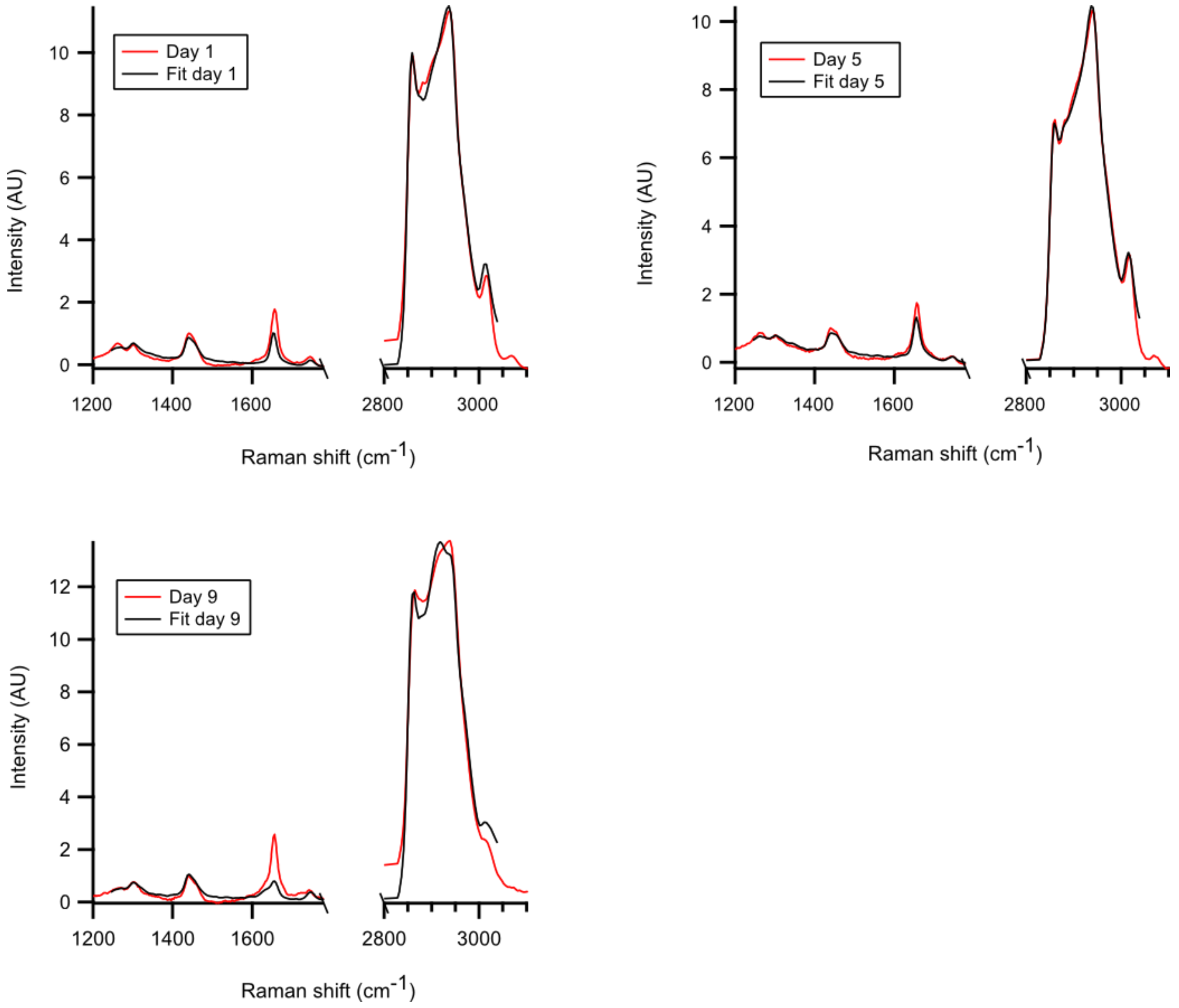

Figure S8. Raman-like spectra and fits for spectra from germinating soybean cotyledons at day 1, day 5, and day 9 post imbibition using a linear combination of the components shown in Figure S7 (top) via eq. 1.

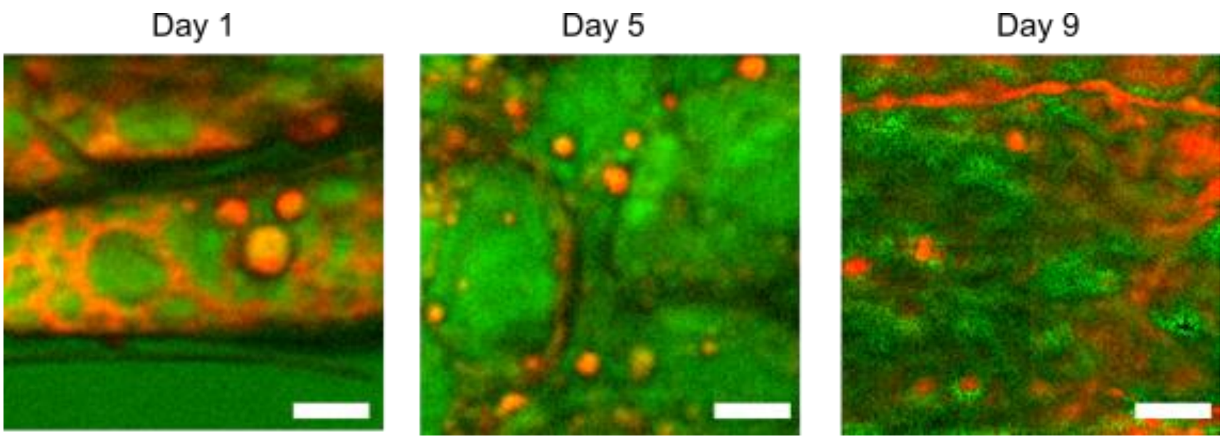

Figure S9. Protein (green) and lipid (red) overlays in the central region of soybean cotyledons at the specified day. Protein contrast was generated by integration of the phenyl ring-breathing mode at 1003 cm^{-1} that is specific for proteins while lipid contrast is created on the CH_2 symmetric mode (2845 cm^{-1}) from hyperspectral Raman-like data of Figure 3 in the main text. Scale bar is 10 µm.

Supplementary Tables

Type	Vibration	Number	Location (cm^{-1})	Location SD (cm^{-1})	FWHM (cm^{-1})
Lorentzian	Phenyl ring breathing	0	997.25	1.92	15
Lorentzian	C-C	1	1077.14	3.99	53
Lorentzian	Amide III / Proteins	2	1218.28	4.64	37
Lorentzian	Amide III	3	1252.02	0.35	35
Lorentzian	CH bending	4	1294.09	2.48	34
Lorentzian	CH2 deformation	5	1435.66	2.63	31
Lorentzian	Amide I / C=C	6	1646.15	1.93	22
Lorentzian	C=O ester for TAGs	7	1735.34	3.01	21

Table S1. Peak fitting parameters for Fingerprint multi-peak fitting of Raman-like spectra. Peak locations were identified by cross-checking against a reference database from (1). FWHM were determined empirically. FWHM were fixed for all spectral fitting. Location SD is the standard deviation of the peak location based on the fitting result from day 1, day 5, and day 9 spectra. The spectral region for each fit was from 972 – 1789 cm^{-1}.

Type	Vibration	Number	Location (cm^{-1})	Location SD (cm^{-1})	FWHM (cm^{-1})
Lorentzian	CH_2 symmetric	0	2846.77	0.10	19
Lorentzian	CH_2 asymmetric	1	2864.51	1.93	29
Lorentzian	CH_2 Fermi	2	2884.78	1.41	34

Lorentzian	CH stretch	3	2905.35	1.08	39
Lorentzian	CH_3	4	2930.15	0.45	40
Lorentzian	Out-of-plane CH_3	5	2962.00	1.10	38
Lorentzian	=CH	6	3007.55	1.56	32

Table S2. Peak fitting parameters for CH multi-peak fitting of Raman-like spectra. Peak locations were identified by cross-checking against a reference database from (1-3). FWHM were seeded with values from (3), and the determined values are in reasonable agreement. FWHM were fixed for all spectral fitting. Location SD is the standard deviation of the peak location based on the fitting result from day 1, day 5, and day 9 spectra. The spectral region for each fit was from 2832 – 3073 cm^{-1}.

Analytical Oil Standard (all from Sigma)	GC mass average chain length	CARS average chain length	Difference	GC mass average # C=C bonds	CARS average #C=C bonds	Difference
Olive	17.7	18.4	-0.7	0.9	0.9	0
Canola	17.9	17.7	0.2	1.3	1.2	0.1
Peanut	18.0	17.1	0.9	1.2	1.0	0.2
Soybean	17.8	17.5	0.3	1.6	1.6	0
Sunflower	17.9	17.5	0.4	1.6	1.4	0.2
Palm	16.9	17.6	-0.7	0.6	0.5	0.1
90% 16-1 TAG: 10% 8-0TAG (v/v)	8.5	8.7	-0.2	0.06	0.06	0
70% 16-1 TAG: 70% 8-0 TAG (v/v)	9.6	10.3	-0.7	0.2	0.2	0
**Root mean square deviation**			**0.5**			**0.1**

Table S3. Calculation of average chain length and # C=C bonds from hyperspectral CARS and GC MS analysis (by the manufacturer) from analytical standard oils or TAG mixtures. The root mean square (RMS) deviation in chain length is ± 0.5 C and the RMS deviation in # C=C bonds is ± 0.1 C=C bonds. Part numbers for the standards are given in the supplemental methods. Note that all plant oils were shipped with a GC certificate that was used to calculate the average chain length and # C=C bonds based on a molar average of the area % of the chromatogram of identified TAGs in the oils.

Supplementary Movie

Movie S1. 320 min live imaging of the flat inside (adxial) part of a soybean cotyledon starting 91 h after imbibition showing intracellular oil body size increase with concurrent disappearance of small oil bodies. Dark regions bordered by lipid reservoirs are vacuoles that grow from day 3-5 during germination.

Supplementary References

1. Movasaghi, Z., S. Rehman, and I. U. Rehman. 2007. Raman spectroscopy of biological tissues. Appl Spectrosc Rev 42:493-541.
2. Gallier, S., K. C. Gordon, R. Jiménez-Flores, and D. W. Everett. 2011. Composition of bovine milk fat globules by confocal Raman microscopy. International Dairy Journal 21:402-412.
3. Potma, E. O., and X. S. Xie. 2003. Detection of single lipid bilayers with coherent anti-Stokes Raman scattering (CARS) microscopy. Journal of Raman Spectroscopy 34:642-650.
